# Effectiveness of diluted povidone-iodine lavage for preventing periprosthetic joint infection: an updated systematic review and meta-analysis

**DOI:** 10.1186/s13018-021-02703-z

**Published:** 2021-09-22

**Authors:** Naomi Kobayashi, Emi Kamono, Kento Maeda, Toshihiro Misumi, Yohei Yukizawa, Yutaka Inaba

**Affiliations:** 1 Department of Orthopaedic Surgery, Yokohama City University Medical Center, 4-57, Urafune-cho, Minami-ku, Yokohama, Japan; 2 Department of Orthopaedic Surgery, Yokohama City University, Yokohama, Japan; 3 Department of Biostatistics, Yokohama City University, Yokohama, Japan

**Keywords:** Periprosthetic joint infection (PJI), Diluted povidone-iodine lavage, Systematic review and meta-analysis

## Abstract

**Background:**

Of the several methods used to prevent surgical site infection (SSI), diluted povidone-iodine (PI) lavage is used widely. However, the clinical utility of PI for preventing periprosthetic joint infection (PJI) remains controversial. The aim of this study was to perform a systematic review and meta-analysis of the utility of dilute PI lavage for preventing PJI in primary and revision surgery.

**Methods:**

This study was conducted in accordance with the PRISMA checklist for systematic reviews and meta-analyses. A comprehensive literature search of PubMed, CINAHL, ClinicalTrials.gov, and Cochrane Library databases was performed. The results are summarized qualitatively and as a meta-analysis of pooled odds ratios with 95% confidence intervals (95% CIs). Heterogeneity of treatment effects among studies was classified as low, moderate, or high, corresponding to *I*^2^ values of < 25%, 25–50%, and > 50%. A random effects model was applied in cases of high heterogeneity; otherwise, the fixed effects model was applied. Subgroup analyses were conducted to identify potential sources of heterogeneity.

**Results:**

After the screening and eligibility assessment process, eight studies were finally extracted for analysis. Overall, the results showed that PI had no significant effect on PJI with ununified control group. However, subgroup analysis of studies with a saline control group revealed an odds ratio of 0.33 (95% CI, 0.16–0.71) for the PI group, suggesting a significant effect for preventing PJI.

**Conclusion:**

The systematic review and meta-analysis of the current literature demonstrates that diluted PI lavage is significantly better than saline solution lavage for preventing PJI.

**Level of evidence:**

Level I, Systematic review and meta-analysis.

## Background

Periprosthetic joint infection (PJI) remains worrisome after total joint arthroplasty. Therefore, methods used to prevent infection should be based on the strongest evidence possible. For instance, perioperative antibiotic prophylaxis [1], skin preparation and draping [2], and some ingenuity in wound closure [3] should be applied. An easy and realistic method is lavage prior to wound closure, particularly methods using antisepsis solutions such as diluted povidone-iodine (PI) or chlorhexidine gluconate (CHG). Indeed, several studies have used antiseptic solutions to prevent PJI.

In terms of preventing surgical site infection (SSI) during general surgery, intraoperative PI is proven to be effective; strong evidence is provided by a meta-analysis of randomized controlled trials conducted approximately 10 years ago [4]. Similarly, another meta-analysis showed that PI lavage significantly reduces SSI after surgery involving spinal instruments [5]. However, PJI is distinct from SSI during general surgery, including abdominal surgery and spine surgery. This is because PJI presents with a particular pathology, including biofilm formation [6] and a specific organism profile [7]; therefore, PI lavage may not have the same effectiveness in preventing PJI as it has in preventing SSI in general surgery. In fact, the results of recent studies on the effectiveness of diluted PI lavage for preventing PJI are controversial [8, 9]. In addition, a recent meta-analysis suggests that diluted PI lavage does not prevent PJI [10]. Thus, a review of the latest evidence is required.

The clinical question of this study is, “Does diluted PI lavage actually reduce the risk of PJI?” The aim of the study was to perform a systematic review and meta-analysis of the current literature concerning the efficacy of diluted PI lavage for preventing PJI in primary and revision surgery.

## Methods

This systematic review and meta-analysis was conducted in accordance with the preferred reporting items for systematic reviews and meta-analyses (PRISMA) checklist for systematic reviews and meta-analyses (http://prisma-statement.org/PRISMAStatement/Checklist).

### Literature search

Multiple comprehensive literature searches of PubMed, Cumulative Index to Nursing and Allied Health Literature (CINAHL), ClinicalTrials.gov, and the Cochrane Library databases were performed on July 13, 2021. Search key words included (“betadine” OR “povidone” OR “povidone-iodine”) AND (“lavage” OR “dilute”) AND (”total hip arthroplasty (THA)” OR “total knee arthroplasty (TKA)” OR “arthroplasty” OR “Periprosthetic joint infection“). An additional manual search was performed to identify other relevant articles or bibliographies.

### Study screening and eligibility assessment

After the first extraction of literature, a first screening was performed by two reviewers. During this screening, the title and abstract were reviewed, and inappropriate literature was excluded. Next, eligibility assessment of full manuscripts was performed by the same two reviewers. The inclusion criteria were as follows: direct comparison between the PI and non-PI lavage groups following total joint arthroplasty (TJA), in which primary or aseptic revision arthroplasty was performed; a PI lavage protocol was used, not a combination protocol with other solutions such as chlorhexidine lavage; the overall infection rate was stated, and when a PI regimen was used for lavage, the article included details of the dosing protocols and the duration of PI application. The exclusion criteria were as follows: non-original clinical research articles, including biomechanical or cadaveric studies, technical notes, letters to the editor, expert opinions, review articles, meta-analyses, and case reports; no full text available; duplicate studies from the same investigation group; and reported follow-up < 3 months.

### Data extraction

Data were extracted from the full text using a piloted form that included the publication date, the study design, type of surgery, number of patients, follow-up length, type of preoperative prophylaxis, type of postoperative prophylaxis, type of intervention (solution type, application method, and volume used), and type of control. Two investigators performed data extraction and reached agreement in all cases.

### Data synthesis and statistical analysis

The results of the systematic review were summarized qualitatively into a meta-analysis of pooled odds ratios with 95% confidence intervals (95% CIs). The analyses were conducted using RevMan 5.3. A *P* value < 0.05 was considered statistically significant. Heterogeneity of treatment effects among studies was evaluated by calculating *I*^2^ and was categorized as low, moderate, or high (*I*^2^ < 25%, 25–50%, and > 50%, respectively). A random effects model was applied in cases of high heterogeneity; otherwise, a fixed effects model was applied. Subgroup analyses were conducted to identify potential sources of heterogeneity. All statistical analyses were performed using Review Manager (RevMan, version 5.3; Copenhagen: The Nordic Cochrane Centre, The Cochrane Collaboration, 2014) (computer program).

### Evaluation of bias risk

Risk of bias in non-randomized studies of interventions (ROBINS-I) was graded by two reviewers per study. The Coleman methodology score (CMS) [11] criteria was also evaluated for research methodological quality by two reviewers per study. Its criteria were slightly modified to suit to the purpose of the present systematic review (Table 1). A test for publication bias was not performed because evaluation of publication bias is typically performed only when at least ten studies are included in a meta-analysis.

**Table 1:** Modified Coleman Methodology Score (CMS) for studies reporting the outcomes of surgery

			Score
Part A: Only one score to be given for each of the seven sections			
1. Study size-number of joint (*N*) (If multiple follow-up, multiply *N* by number of times subjects followed up)		● >300	10
		● 200–300	7
		● 100–200	4
		● <100	0
2. Mean follow-up (months)		● ≥12	5
		● ≥3,and <12	2
		● <3	0
3. Number of different surgical procedures included in each reported outcome. More than one surgical technique may be assessed but separate outcomes should be reported		● One surgical procedure only	10
		● More than one surgical procedure, but >90% of subjects undergoing the one procedure	7
		● Not stated, unclear or °90% of subjects undergoing the one procedure	0
4. Type of study		● Randomized control trial	15
		● Prospective cohort study	10
		● Retrospective cohort study	0
5. Diagnostic certainty Compliance with diagnostic guidelines or their content for PJI		● In all	5
		● in >80%	3
		● in <80%, no, NS or unclear	0
6. Description of surgical procedure given		● Adequate (technique stated and necessary details of that type of procedure given)	5
		● Fair (technique only stated without elaboration)	3
		● Inadequate, not stated or unclear	0
7. Description of preoperative and postoperative prophylaxis		● Well described	10
		● Fair (technique only stated without elaboration)	5
		● Protocol not reported	0
Part B: Scores may be given for each option in each of the three sections if applicable			
1. Outcome criteria (If outcome criteria are vague and do not specify subjects’ sporting capacity, score is automatically 0 for this section)		● Outcome measures clearly defined	2
		● Timing of outcome assessment clearly stated (e.g., at best outcome after surgery or at follow-up)	2
		● Use of outcome criteria that has reported good reliability	3
		● Use of outcome with good sensitivity	3
2. Procedure for assessing outcomes		● Subjects recruited (results not taken from surgeons’ files)	5
		● Investigator independent of surgeon	4
		● Written assessment	3
		● Completion of assessment by subjects themselves with minimal investigator assistance	3
3. Description of subject selection process		● Selection criteria reported and unbiased	5
		● Recruitment rate reported: >80% or	5
		● <80%	3
		● Eligible subjects not included in the study satisfactorily accounted for or 100% recruitment	5

## Results

### Literature screening and identification

**Fig. 1:**
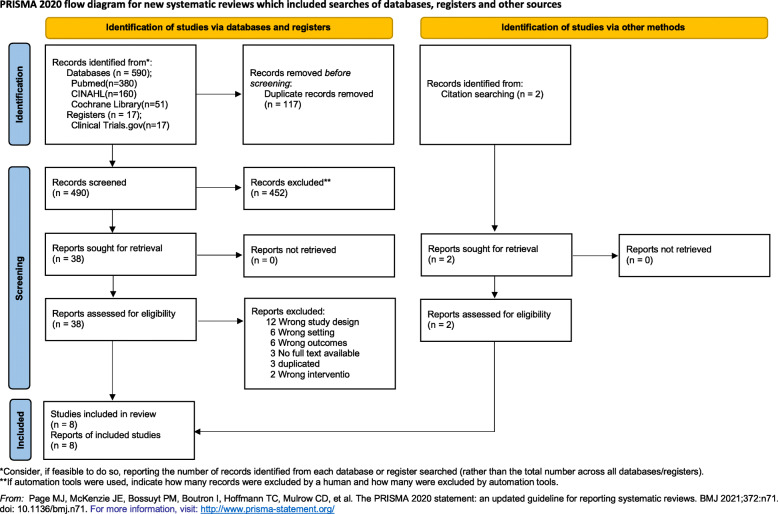
Preferred Reporting Items for Systematic Reviews and Meta-Analyses (PRISMA) flow diagrams for identification and selection of studies to be included in the meta-analysis

Figure 1 shows the detailed flow and the number of screened publications. The initial electronic database search identified 590 studies. Two additional manual search records were added from other sources. Thus, 490 studies were screened after removing 117 duplicates. After the screening and eligibility assessment process, eight publications [8, 9, 12–17] were included in the analysis.

### Study and treatment characteristics

Table 2 summarizes the study demographics. Only one of the eight studies was a randomized controlled trial; the other seven were retrospective cohort studies. Table 3 summarizes the treatment characteristics in each study. In three studies, we found that the control group was set as “lavage with saline,” whereas chlorhexidine gluconate was used in two studies. No information was provided by three studies.

**Table 2:** Study demographics and characteristics

Author	Year	Study design	Type of Surgery		Sample Size (Overall)		Follow-up Length	CMS
					PI	non PI		
Brown et al	2012	RCS	THA,TKA	Primary	688	1862	3 mo	71
Frisch et al	2017	RCS	THA,TKA	Primary	253	386	12 mo	35
Fleischman et al	2018	RCS	TKA	Primary	2124	7665	3 mo	35
Hart et al	2019	RCS	THA,TKA	Revision	540	1835	3,12 mo	52
Hernandez et al	2019	RCS	THA,TKA	Primary	3067	7214	3,12 mo	53
Calkins et al	2020	RCT	THA,TKA	Revision	223	234	3 mo	80
Driesman et al	2020	RCS	THA,TKA	Primary	1227	1159	3,12 mo	73
Slullitel et al	2020	RCS	THA,TKA,HRs	Primary	2268	2268	3 mo	67

*Abbreviations*: *PI* Povidone-Iodine, *RCT* randomized controlled trials, *RCS* retrospective cohort study, *THA* total hip arthroplasty, *TKA* total knee arthroplasty, *CMS* Coleman Methodology Score

**Table 3:** Treatment characteristics in each study

Author and year	Year	Preoperative Prophylaxis	Intraoperative Intervention Treatment	Intraoperative Control Treatment	Postoperative Prophylaxis
Calkins et al	2020	NR	500 ml dilute betadine solution for 3min with of the consisted of 17.5ml PI and 500ml NaCl after implantation, followed by 1L NaCl. Also, the wound edges were painted with 10% PI with a sponge stick	1-L pulsatile lavage of normal saline irrigation	NR
Driesman et al	2020	Ancef or vancomycin (depending on MRSA risk)	500 ml of the dilute betadine solution made of 17.5ml PI and 500ml NaCl for 3min after implantation	agents chlorhexidine irrigation	in-wound antibiotics in the form of 2 g of vancomycin powder
Hernandez et al	2019	Cefazolin (vancomycin or clindamycin if allergic).	dilute PI solution for 3 minutes	NR	Cefazolin (vancomycin or clindamycin if allergic) for 24 hours
Slullitel et al	2020	cefazolin or vancomycin if allergic	one surgeon used a 115ml nonsterile bottle of 10% PI diluted in 500ml of sterile saline (0.45%) for 3 minutes. 9 surgeons used a 22.5-mL sterile solution pouch of 10% PI diluted in 250-500 mL of saline (0.2%-0.35%) for 1-3 minutes.	500ml of sterile saline solution before wound closure	cefazolin or vancomycin if allergic for 24 hours
Hart et al	2019	NR	1L of sterile 0.25% PI 3 minutes followed by irrigation with normal saline solution prior to closure.	NR	IV for 24 hours
Fleischman et al	2018	NR	Intraoperative dilute betadine irrigation	NR	systemic antibiotic prophylaxis
Frisch et al	2017	vancomycin and cefazolin (gentamicin if allergic)	intraoperative irrigation with 0.9% saline followed by a 2-minute soak with <2% dilute PI which was washed out entirely before closure	intraoperative irrigation with 0.9% saline and periodic 0.05% CHG solution followed by a final 1-minute soak in CHG with immediate closure afterward	cefazolin was given for 2 doses to be discontinued within 24 hours
Brown et al	2012	cefazolin within 1 hour.	500mL 0.35% PI solution for 3min after implantation, followed by 1L 0.9% NaCl pulsatile lavage with PI painting	1L isotonic sodium chloride solution irrigation	Cefazolin (vancomycin or clindamycin if allergic) for 24 hours

*Abbreviations*: *PI* Povidone-Iodine, *IV* intravenous, *MRSA* methicillin-resistant Staphylococcus aureus, *NR* not reported

### Individual study results and synthesis of results

**Fig. 2:**
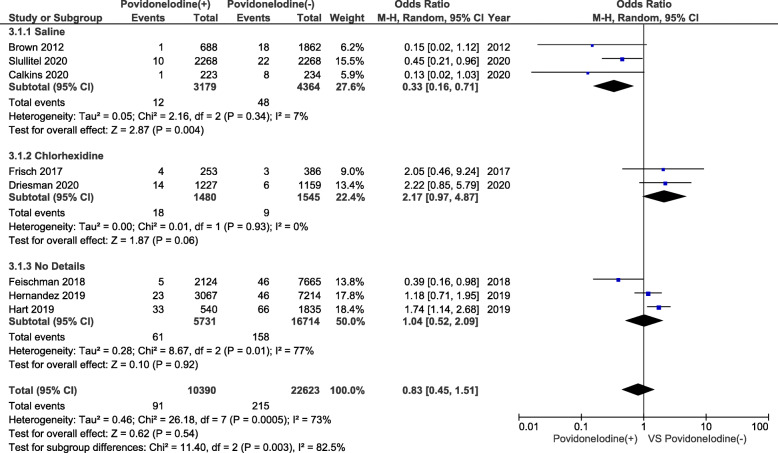
Forest plot of the random effects model showing the odds ratio and 95% confidence intervals for PI lavage (+) compared with those for PI lavage (−)

Table 4 summarizes the PJI rates in each study. In total, 10,390 subjects were identified as belonging to the PI lavage group, and 22,623 subjects were identified as belonging to the non-PI lavage group. In the PI lavage group, 91 were identified as having PJI compared with 215 in the non-PI lavage group. Figure 2 shows the results of the meta-analysis. In studies that used a saline control group, the odds ratio for PI lavage was 0.33 (95% CI, 0.16–0.71; *P* = 0.004); thus, the risk of PJI was significantly reduced. In studies that used a CHG control group, the odds ratio for PI lavage was 2.17 (95% CI, 0.97–4.87; *P* = 0.06); in this case, the difference was not significant. In studies that provided no detailed information about the control groups, the odds ratio of PI lavage was 1.04 (95% CI, 0.52–2.09; *P* = 0.92), i.e., no significant difference. Overall, the odds ratio for PI lavage was 0.83 (95% CI, 0.45–1.51; *P* = 0.54), suggesting that PI lavage has no significant effect on the risk of PJI, although the control group was not unified in this setting.

**Table 4:** Results of each individual study

Author	Year	PI lavage (+) Patients	PI lavage (-) Patients	PJI rate in PI lavage (+) Patients		PJI rate in PI lavage (-) Patients	
Brown et al	2012	688	1862	0.1%	(1/688)	1.8%	(18/1862)
Frisch et al	2017	253	386	1.6%	(4/253)	0.8%	(3/386)
Fleischman et al	2018	2124	7665	0.2%	(5/2124)	0.6%	(46/7665)
Hart et al	2019	540	1835	6.1%	(33/540)	3.6%	(66/1835)
Hernandez et al	2019	3067	7214	0.7%	(23/3067)	0.6%	(46/7214)
Calkins et al	2020	223	234	0.4%	(1/223)	3.4%	(8/234)
Driesman et al	2020	1227	1159	0.6%	(7/1227)	0.4%	(5/1159)
Slullitel et al	2020	2268	2268	0.8%	(10/2268)	1.0%	(22/2268)

*Abbreviations*: *PI* Povidone-Iodine, *PJI* periprosthetic joint infection

### Risk of bias and quality assessment

**Fig. 3:**
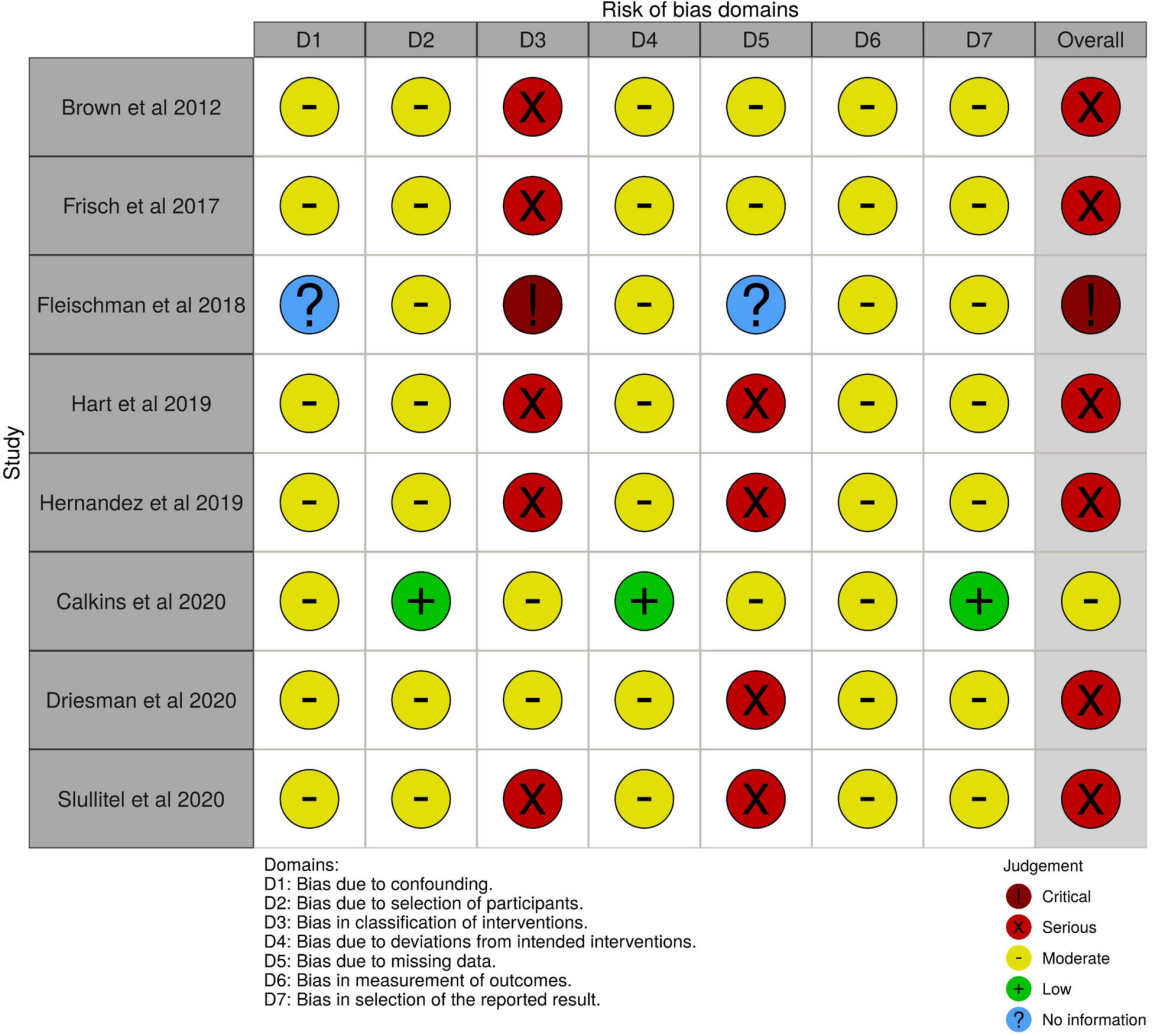
Evaluating the risk of bias using risk of bias in non-randomized studies of interventions (ROBINS-I)

Figure 3 summarizes the risk of bias. In one study (Calkins et al. [9]), the overall risk of bias was “moderate.” In another study (Fleischman et al. [14]), the overall risk of bias was “critical.” For the remaining six studies, the overall risk of bias was “serious.” The CMS ranged from 35 to 80 within component studies (mean: 58.25, SD: 17.23, median: 60) (Tables 2 and 5).

**Table 5:** Coleman methodology score (CMS): mean, SD, range, and median values for each component element of CMS

Components of CMS	Mean	SD	Range			Median
Study size (10)	9.25	1.39	7	–	10	10
Mean duration of follow-up (5)	3.88	1.55	2	–	5	5
Number of different surgical procedures included in each reported outcome (10)	4.63	5.04	0	–	10	3.5
Type of study (15)	1.88	5	0	–	15	0
Diagnostic certainty (5)	1.88	1.55	0	–	3	3
Description of surgical procedure (5)	4.29	3.45	0	–	10	5
Description of postoperative prophylaxis (10)	6.88	3.72	0	–	10	7.5
Outcome measures (10)	7.25	3.28	0	–	10	7
Outcome assessment (15)	11.00	5.24	3	–	15	13
Selection process (15)	7.88	4.19	0	–	13	10
Total	58.25	17.23	35	–	80	60

## Discussion

The most important result in this study is that dilute PI lavage is significantly more effective against PJI than saline lavage. Although the combined results of all studies suggest that PI lavage has no significant effect, careful consideration of the negative control conditions used in each study led us to conclude that dilute PI lavage is significantly more effective in preventing PJI than saline lavage in routine surgical procedures.

The oldest study, conducted by Brown et al. [12], was a retrospective cohort study with a total of 2540 consecutive patients receiving total joint arthroplasty; they reported a significantly lower rate of infection in the dilute PI lavage group than in the control saline lavage group. Also, a recent large retrospective cohort study reported a lower rate of infection in a PI group than in a saline lavage group when the groups were propensity-matched [17]. Similarly, a study by Calkin revealed that dilute PI lavage significantly reduced occurrence of acute PJI after aseptic revision of TKA and THA [9]. This study had the lowest risk of bias due to its randomized controlled trial design and the inclusion of a defined negative control, saline lavage. By contrast, two another retrospective cohort studies conducted by the same group using a relatively large number of samples concluded that dilute PI lavage does not reduce the risk of reoperation for infection after both primary and revision THA and TKA [8, 16]. Thus, there was no consensus regarding the routine use of dilute PI lavage for the prevention of PJI.

We identified only one previous meta-analysis study examining the effectiveness of dilute PI lavage for the prevention of PJI; this was a systematic review study by Kim et al. [10]. They found no difference in the overall postoperative infection rates between the PI and non-PI lavage groups. While our overall results agree with these results, our sub-analysis of studies that reported saline solution controls clearly shows that PI lavage reduces rates of PJI significantly. There are several possible reasons for this discrepancy. First, we included very recent publications from 2020, which showed positive results for PI lavage. Second, we performed sub-analysis excluding studies that used CHG as a control or that provided no detailed information about the control solution. A retrospective study by Hart et al. showed negative effect of PI lavage for preventing PJI in large cohort of revision arthroplasty. This study did not clearly define control group protocol that was just “no use of PI lavage.” In addition, the use of PI lavage was decided only by surgeon’s discretion that should arise severe selection bias. Studies that compared PI and CHG failed to show an advantage of PI. Indeed, the study by Driesman et al. compared PI and CHG lavage as preventive measures for PJI [13], but found no differences in their effectiveness. Because the study was conducted to show the “non-inferiority” of CHG compared with PI, we could not use this study to investigate the effectiveness of PI. Indeed, we found a significant negative effect in our sub-analysis using CHG control groups. Thus, our overall analysis identified high heterogeneity: *I*^2^ = 73%. Similarly, we excluded studies that did not provide detailed information about control groups. This was the case for three retrospective cohort studies, which were excluded from subgroup analysis. Thus, subgroup analysis of studies that included a saline control showed low heterogeneity *I*^2^ = 7%.

Several recent studies were excluded from this systematic review during eligibility assessment, some of which reported evidence supporting PI utility. Nazal et al. reported that treating sterile water splash basins with dilute PI (0.02% solution) eliminates intraoperative contamination of such splash basins during total joint arthoplasty [18]. This may contribute indirectly to reducing the risk of PJI. Cichos et al. conducted an *in vitro* study to compare the effectiveness of PI, CHG gluconate, and vancomycin with respect to minimal inhibitory concentrations (MIC) and time to death of multiple bacteria [19]. They showed that all bacterial isolates tested were killed only by PI and that PI killed all bacteria tested immediately on contact; exposure time was not the key factor. On the other hand, a negative finding was that PI had a chondrotoxic effect on the superficial cartilage layer [20].

It should be noted that all except one of the publications analyzed herein were retrospective in design; therefore, the risk of bias was high (“serious”) in seven of the eight studies. Further studies with a lower risk of bias (i.e., a prospective randomized design with strict negative controls) are needed to support the evidence that PI is effective in preventing PJI. In addition, alternatives to PI solution lavage, such as CHG solution lavage [21] or vancomycin powder [22], should be investigated for their ability to eradicate PJI.

## Conclusion

This systematic review and meta-analysis of the current literature demonstrates that diluted PI lavage is significantly better than saline solution lavage for preventing PJI. We recommend diluted PI lavage (0.35%) be used to prevent PJI rather than saline solution lavage.

## Data Availability

The datasets used and analyzed during the current study are available from the corresponding author on reasonable request.

## Abbreviations

SSI: Surgical site infection
PI: Povidone-iodine
PJI: Periprosthetic joint infection
CHG: Chlorhexidine gluconate
THA: Total hip arthroplasty
TKA: Total knee arthroplasty
TJA: Total joint arthroplasty
ROBINS-I: Risk of bias in non-randomized studies of interventions
